# Correlation between the incidence of inguinal hernia and risk factors after radical prostatic cancer surgery: a case control study

**DOI:** 10.1186/s12894-024-01493-w

**Published:** 2024-06-22

**Authors:** An-Ping Xiang, Yue-Fan Shen, Xu-Feng Shen, Si-Hai Shao

**Affiliations:** 1 Department of Urology, The First People’s Hospital of Huzhou, #158, Square Road, Huzhou, 313000 China; 2 Department of Urology, Huzhou Key Laboratory of Precise Diagnosis and Treatment of Urinary Tumors, Huzhou, 313000 China

**Keywords:** Prostate cancer, Inguinal hernia

## Abstract

**Objective:**

The incidence of recurrent hernia after radical resection of prostate cancer is high, so this article discusses the incidence and risk factors of inguinal hernia after radical resection of prostate cancer.

**Methods:**

This case control study was conducted in The First People’s Hospital of Huzhou clinical data of 251 cases underwent radical resection of prostate cancer in this hospital from March 2019 to May 2021 were retrospectively analyzed. According to the occurrence of inguinal hernia, the subjects were divided into study group and control group, and the clinical data of each group were statistically analyzed, Multivariate Logistic analysis was performed to find independent influencing factors for predicting the occurrence of inguinal hernia. The Kaplan-Meier survival curve was drawn according to the occurrence and time of inguinal hernia.

**Results:**

The overall incidence of inguinal hernia after prostate cancer surgery was 14.7% (37/251), and the mean time was 8.58 ± 4.12 months. The average time of inguinal hernia in patients who received lymph node dissection was 7.61 ± 4.05 (month), and that in patients who did not receive lymph node dissection was 9.16 ± 4.15 (month), and there was no significant difference between them (*P* > 0.05). There were no statistically significant differences in the incidence of inguinal hernia with age, BMI, hypertension, diabetes, PSA, previous abdominal operations and operative approach (*P* > 0.05), but there were statistically significant differences with surgical method and pelvic lymph node dissection (*P* < 0.05). The incidence of pelvic lymph node dissection in the inguinal hernia group was 24.3% (14/57), which was significantly higher than that in the control group 11.8% (23/194). Logistic regression analysis showed that pelvic lymph node dissection was a risk factor for inguinal hernia after prostate cancer surgery (OR = 0.413, 95%Cl: 0.196–0.869, *P* = 0.02). Kaplan-Meier survival curve showed that the rate of inguinal hernia in the group receiving pelvic lymph node dissection was significantly higher than that in the control group (*P* < 0.05).

**Conclusion:**

Pelvic lymph node dissection is a risk factor for inguinal hernia after radical resection of prostate cancer.

Prostate cancer is a common malignant tumor in urology, which occurs in the prostate epithelial tissue, There are an average of 190,000 new cases of prostate cancer each year and about 80,000 deaths worldwide each year [1, 2]. In recent years, the incidence of prostate cancer has increased year by year, seriously affecting the health and quality of life of patients [3]. Worldwide, the incidence of prostate cancer is second only to lung cancer, and its death rate ranks 7th among male cancer causes [4]. Radical resection of prostate cancer (RP) is the main means for the treatment of prostate cancer, and the surgical methods are generally divided into open radical resection of prostate cancer (RRP) and minimally invasive radical resection of prostate cancer, the latter including laparoscopic radical resection of prostate cancer (LRP) and robot-assisted laparoscopic radical resection of prostate cancer (RALP) [5–7].

Inguinal hernia (IH) is a relatively common disease in clinic, which is caused by increased abdominal pressure, thinning of abdominal wall, and bulging of abdominal organs. Inguinal hernias include direct hernias, oblique hernias and femoral hernias [8]. At the onset, lumps protruding outward from the inguinal region can be seen. If the intestines cannot return to the abdominal cavity in time, it is easy to cause intestinal necrosis, intestinal obstruction, intestinal perforation and other complications, which may endanger the life safety of patients in severe cases [9, 10].

With the extensive development of radical resection of prostate cancer in various hospitals, the problem of postoperative inguinal hernia has gradually attracted the attention of urologists. The previously reported incidence of IH after radical prostate cancer surgery was approximately 13.7% [11]. A study by Nagatani S et al. showed that the incidence of inguinal hernia after radical prostate cancer surgery was 7-21%, most of which occurred within 2 years after surgery [12]. A study by Stranne J et al. showed that the cumulative risk of IH occurrence within 48 months in open radical resection for prostate cancer group and non-surgical group was 12.2% and 5.8%, respectively [13]. Most cases of IH require surgery due to pain, discomfort, and incarceration and are considered an advanced complication of radical resection of prostate cancer. The adhesion after radical resection of prostate cancer also increases the difficulty of hernia repair. Therefore, urologists need to be concerned not only about the risk of urinary incontinence and erectile dysfunction after radical resection of prostate cancer, but also about the occurrence of IH.

In recent 10 years, many scholars around the world have studied the risk factors of inguinal hernia after radical prostate cancer surgery. Currently, most of the studies believe that anastomotic stenosis, previous history of inguinal hernia, and patent processus vaginalis are risk factors, However there is no consensus on the risk of lymph node dissection. For example, Niitsu H et al. believed that pelvic lymph node dissection during radical prostate cancer operation might damage the pectineal foramina, thereby increasing the risk of inguinal hernia [14]. Contrary to the results of Johan Stranne’s study, the author suggested that previous incidence of inguinal hernia and advanced age increased the risk of inguinal hernia after radical prostate cancer surgery, and pelvic lymph node dissection was not a significant risk factor [15]. There is also no consistent conclusion on the influence of BMI, age and surgical method.

Therefore, in order to further investigate the risk factors of inguinal hernia after radical prostate cancer surgery, especially the correlation between pelvic lymph node dissection and inguinal hernia, this study was conducted. This study retrospectively analyzed the clinical data of 251 patients who underwent radical resection of prostate cancer in our hospital from March 2019 to May 2021, and investigated the risk factors of postoperative inguinal hernia. It is reported as follows:

## Methods

### Research objectives

The objective of this study was to explore the incidence and risk factors of inguinal hernia after radical resection of prostate cancer, which provides reference for further research and guide the clinician to choose the appropriate surgical method according to the patient’s condition.

### Research methods

The patient was also examined by B-ultrasound every 3 months at the outpatient PSA review to verify the occurrence of inguinal hernia. The subjects were divided into the inguinal hernia group (study group) and the non-inguinal hernia group (control group), If the diagnosis of inguinal hernia occurred, the follow-up was completed, and the type and time of inguinal hernia were recorded; otherwise, the follow-up was 2 years, and the relevant clinical parameters of each group were statistically analyzed (age, BMI, hypertension, diabetes mellitus, PSA value, previous abdominal operations, operation methods, operative approach, pelvic lymph node dissection)and the correlation between these parameters and the occurrence of inguinal hernia was analyzed, and the risk factors of inguinal hernia were found by Logistic regression analysis. According to the occurrence and time of inguinal hernia, Kaplan-Meier survival curve was drawn to compare the differences between the two groups.

The content of this study has been approved by the Ethics Committee of our hospital(approval number, 2,018,137). All patients signed informed consent forms. This is the protocol was registered on the Chinese Clinical Trial Registry. The study is planned to begin in mid-March 2019 and is planned to end by May 2021.

### Inclusion criteria

Patients who received radical surgery for prostate cancer in Huzhou First People’s Hospital from March 2019 to May 2021; PSA was reviewed every 3 months after surgery, and check the inguinal area for protruding masses. Complete the 2-year follow-up plan.

### Exclusion criteria

Patients with inguinal hernia before operation; patients with prior inguinal hernia surgery.

### Statistical methods

SPSS 21.0 statistical software was used for statistical processing, the research data followed normal distribution, and the measured data were represented by X ± S. *P* < 0.05 was considered statistically significant.

## Results

From March 2019 to May 2021, 318 cases of radical prostatectomy were performed in our hospital, during the follow-up period, a total of 28 cases died of other diseases, a total of 39 cases were lost to follow-up or clinical data were incomplete, and a total of 251 cases were finally followed up. There were no significant differences in age, BMI, hypertension, diabetes, PSA, previous abdominal operations and operative approach between the two groups (*P* > 0.05), while there were significant differences in surgical method and pelvic lymph node dissection (*P* < 0.05). The incidence of pelvic lymph node dissection in the inguinal hernia group 24.3% (14/57) was significantly higher than that in the control group 11.8% (23/194). See Table 1 for details.

**Table 1:** Clinically relevant parameters

Parameter	Hernia group (*N* = 37)	No-hernia group (*N* = 214)	Statistic	*P* value
Age(years)	67.88 ± 9.11	69.23 ± 6.63	T=-0.86	0.391
BMI(kg.m^2^)	22.39 ± 3.66	23.06 ± 3.58	T=-1.042	0.299
Hypertension			χ2 = 1.226	0.268
Yes	19	89		
No	18	125		
Diabetes mellitus			χ2 = 0.627	0.429
Yes	12	56		
No	25	158		
PSA(ng/ml -)	14.31 ± 4.58	12.84 ± 4.6	T = 1.67	0.096
previous abdominal operations			χ2 = 1.042	0.307
Yes	6	51		
No	31	163		
Operation methods			χ2 = 4.85	0.028
RRP	15	50		
LRP	22	164		
Operative approach			χ2 = 0.32	0.57
Transperitoneal	5	37		
Extraperitoneal	32	177		
Pelvic lymph node dissection			χ2 = 5.65	0.017
Yes	14	43		
No	23	171		

Multivariate Logistic regression analysis of risk factors showed that pelvic lymph node dissection was a risk factor for inguinal hernia after prostate cancer surgery (OR =0.413, 95%Cl: 0.196-0.869, *P* = 0.02). There was no statistical significance in age, BMI, hypertension, diabetes, PSA value, previous abdominal operations, operation method, operative approach were not risk factors for inguinal hernia (*P* > 0.05). See Table 2 for details.

**Table 2:** Multivariate Logistic regression analysis of inguinal hernia after prostate cancer surgery

Parameter	OR	95% confidence interval for EXP(B)		*P* value
		Lower limit	Upper limit	
Pelvic lymph node dissection	0.413	0.196	0.869	0.02
Age	1.009	0.948	1.074	0.77
BMI	1.047	0.923	1.189	0.473
Hypertension	0.792	0.369	1.697	0.548
Diabetes mellitus	1.09	0.454	2.618	0.848
PSA	0.936	0.866	1.012	0.096
Previous abdominal operations	2.038	0.758	5.481	0.158
Operation methods	0.464	0.204	1.053	0.066
Operative approach	1.105	0.385	3.173	0.853

**Fig. 1:**
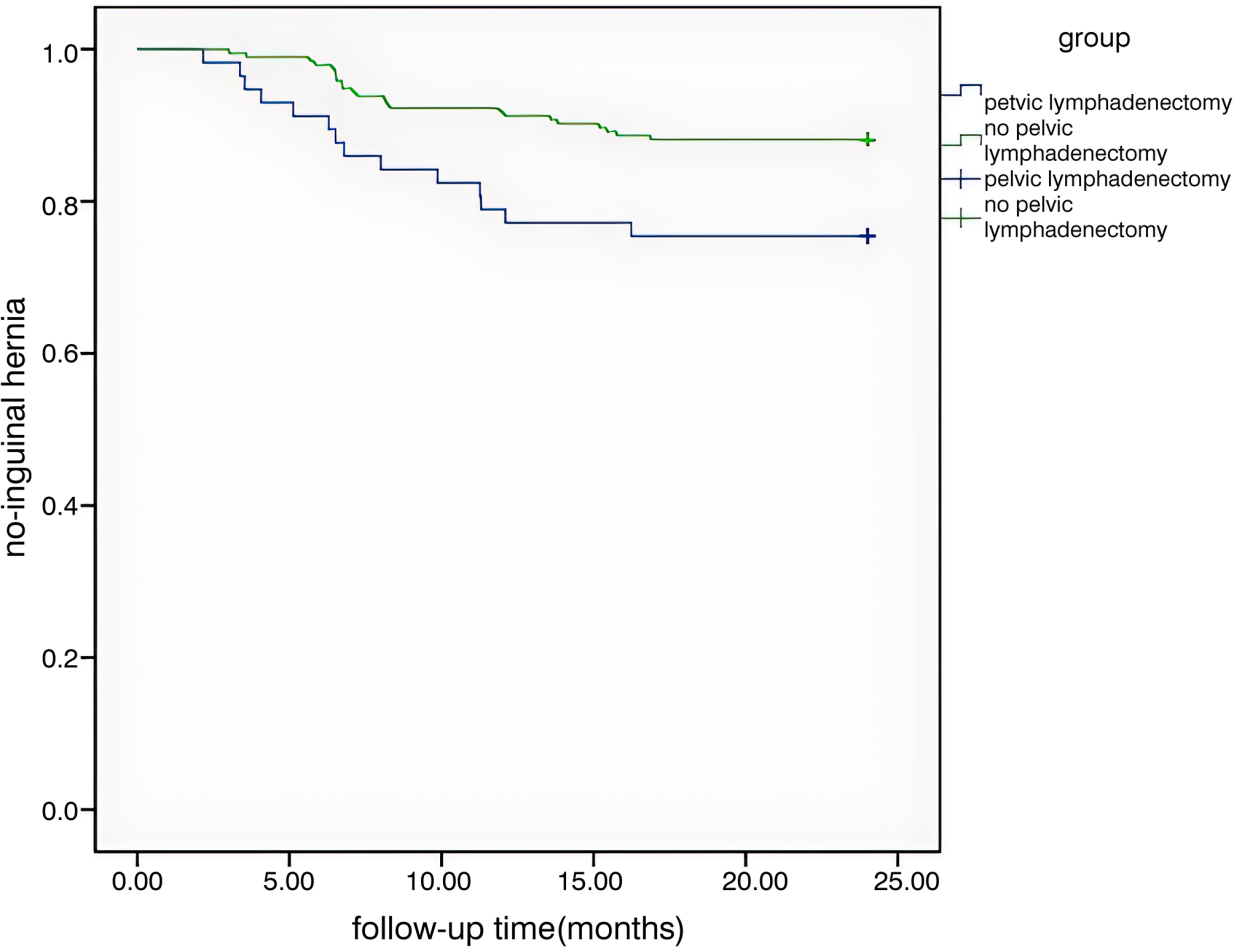
Survival curve of pelvic lymph node dissection and inguinal hernia (month)

The cases of inguinal hernia were grouped according to whether or not they had received pelvic lymph node dissection. The incidence and time of inguinal hernia in the two groups were recorded, and the Kaplan-Meier survival curve was drawn. The overall incidence of inguinal hernia after radical resection of prostate cancer was 14.7% (37/251), There were 26 cases with indirect hernia, accounting for 70.2% (26/37), 21.6% (8/37) with direct hernia, 8.2% (3/37) with oblique hernia and direct hernia, and the mean time of occurrence was 8.58 ± 4.12 months. The average time of inguinal hernia was 7.61 ± 4.05 (month) for those who received lymph node dissection and 9.16 ± 4.15 (month) for those who did not receive lymph node dissection, and there was no significant difference between them (*P* > 0.05). The incidence of inguinal hernia in the group receiving pelvic lymph node dissection was significantly higher than that in the control group (*P* < 0.05). See Fig. 1 for details.

## Discussion

In recent years, the incidence of prostate cancer has increased year by year, seriously affecting the health and quality of life of patients, the complications after radical prostate cancer surgery mainly include urinary incontinence and sexual dysfunction, but inguinal hernia is also one of the common complications [16]. Liu L et al. found that open radical resection for prostate cancer technique and advanced patient age, especially those over 80 years old, are associated with a higher incidence of IH. Appropriate prophylaxis during surgery should be evaluated in high-risk patients [17].In some regional studies, low BMI has been identified as a risk factor for IH, and the risk threshold for BMI has not been determined, which is about BMI < 25 kg/m2 [18]. However, a number of studies have found that low BMI does not increase the risk of postoperative IH [19, 20]. At present, there is no uniform conclusion on the risk of IH between open radical resection for prostate cancer and laparoscopic radical prostatectomy. The study of Alder R scholars believed that the incidence of IH after laparoscopic radical prostatectomy was relatively low [21], while Otaki T’s study shows that the incidence of IH after laparoscopic radical prostatectomy is 7.3% and that of open radical resection for prostate cancer is 8.4%, showing no statistical difference between them [20]. There is no consensus on whether pelvic lymph node dissection is a risk factor for inguinal hernia [14, 15]. In short, the specific mechanism of inguinal hernia after radical prostate cancer surgery is unclear.

This study retrospectively analyzed the clinical data of 251 cases treated in our hospital, and found that the overall incidence of inguinal hernia was 14.7% (37/251), which was consistent with most of the current research results. We also found that the average time of occurrence of inguinal hernia after surgery was 8.58 ± 4.12 months, which provided certain guidance for our postoperative follow-up time.

In this study, through Logistic multivariate analysis, it was found that pelvic lymph node dissection was a risk factor for inguinal hernia after prostate cancer surgery (OR = 0.413, 95%Cl: 0.196–0.869, *P* = 0.02). There was no statistical significance in age, BMI, hypertension, diabetes, PSA value, previous abdominal operations, operation method, operative approach and the occurrence of inguinal hernia after prostate cancer surgery (*P* > 0.05),but there were statistically significant differences with surgical method and pelvic lymph node dissection (*P* < 0.05). Therefore, the advantages and disadvantages of pelvic lymph node dissection should be reasonably evaluated for low-medium-risk prostate cancer patients, so as to avoid the occurrence of inguinal hernia. By drawing Kaplan-Meier survival curve, it was found that the rate of inguinal hernia in the group receiving pelvic lymph node dissection was significantly higher than that in the control group. Some studies believe that pelvic lymph node dissection during radical resection of prostate cancer operation will cause postoperative scar contraction in the inguinal region, resulting in an increase in abdominal pressure outward and downward, resulting in an increase in the incidence of inguinal hernia. Lodding P designed a comparative study between the group of radical resection of prostate cancer plus pelvic lymph node dissection, the group of pelvic lymph node dissection and the group without operation. They found that the incidence of inguinal hernia in the three observation groups was 13.6%, 7.6% and 3.1%, respectively, and the difference between the prostatectomy group and the group without operation was statistically significant. There was no significant difference between the group and pelvic lymph node dissection group. This result implies that pelvic lymph node dissection is an important factor in the development of inguinal hernia [22]. Another Sun M study compared the incidence of inguinal hernias after radical prostate cancer surgery and pelvic lymph node dissection alone, and showed that the risk of inguinal hernias increased by 6.8% and 7.8% at 5 and 10 years, respectively, in the radical prostate cancer resection group compared with the pelvic lymph node dissection group [23]. Niitsu H et al. believed that pelvic lymph node dissection during radical resection of prostate cancer might damage the pectineal foramina, while inguinal hernia originated from the defective pectineal foramina [14].

Shimbo M et al. found that due to prostatectomy and vesicourethral anastomosis, preoperative and postoperative sagittal MRI images showed that the rectovesical excavation (RE) was moved downward by about 2 to 3 cm [24]. Accordingly, they speculated that due to the displacement of RE, the peritoneum and vas deferens after urethrovesical anastomosis were pulled, which further pulled the opening of the inner ring and caused it to shift medially, which led to the occurrence of postoperative IH. Based on this theory, many scholars have prevented the occurrence of hernia after operation by reducing the tension of peritoneum and vas deferens at the inner ring and ligation and rupture of sheathing process. Several other articles have reported the role of preserving the retropubic space (RS) in preventing IH after radical resection of prostate cancer. Chang KD et al. found that robot-assisted laparoscopic radical prostatectomywith retained Retzius space significantly reduced the incidence of postoperative IH compared with standard robot-assisted laparoscopic radical prostatectomy [25]. In addition, the study of Matsubara et al. also showed that compared with standard open radical resection for prostate cancer, the incidence of IH after transperineal radical resection of prostate cancer with retained anatomical structures such as the Retzius space was lower [26]. Therefore, urological surgeons can take some effective measures in the operation to prevent the recurrence of inguinal hernia.

In this study, we identified risk factors for inguinal hernia after pelvic lymphadenectomy for prostate cancer. Other risk factors such as age, BMI, hypertension, diabetes mellitus, PSA value, history of abdominal surgery, operative method, operative approach were not significant in multivariate analysis, which was inconsistent with the results of Iwamoto H et al [27]. They found that dilatation of the right internal inguinal ring and different manipulation of the medial peritoneal incision of the ventral femoral ring were independent risk factors for IH after laparoscopic radical prostatectomy. The reason why postoperative IH occurs more often on the right side is not known. Alder R et al. found that the incidence of IH after open radical prostate cancer treatment was significantly higher than laparoscopic radical prostate cancer treatment [21], but our study did not show a difference between the two groups, possibly due to the small number of cases included in open radical prostate surgery.

In summary, the incidence of inguinal hernia after radical prostate cancer surgery is relatively high, and the specific cause is still unclear. Our study shows that pelvic lymph node dissection is a risk factor for inguinal hernia.

### Limitations

The sample size of this study is small, and it belongs to a single-center study, so the representativeness of the research conclusions may not be strong. This time, we followed up the samples for 2 years, which was not long enough and may have overlooked the real incidence of inguinal hernia. In addition, this study is a retrospective study, and the clinical parameters observed are not very comprehensive, which may ignore the influence of other factors on the IH. Because our data is derived from clinical data, some data cannot be detected. These problems need further study by more scholars.

## Data Availability

We cannot provide and share our datasets in publicly available repositories because of informed consent for participants as confidential patient data. Data may be obtained from the corresponding author upon reasonable request.
